# A systematic review of multidomain and lifestyle interventions to support the intrinsic capacity of the older population

**DOI:** 10.3389/fmed.2022.929261

**Published:** 2022-07-15

**Authors:** Roberta Bevilacqua, Luca Soraci, Vera Stara, Giovanni Renato Riccardi, Andrea Corsonello, Giuseppe Pelliccioni, Fabrizia Lattanzio, Sara Casaccia, Johanna Möller, Rainer Wieching, Toshimi Ogawa, Suichiro Watanabe, Keisuke Kokobun, Izumi Kondo, Eiko Takano, Elvira Maranesi

**Affiliations:** ^1^Scientific Direction, IRCCS INRCA, Ancona, Italy; ^2^Unit of Geriatric Medicine, IRCCS INRCA, Cosenza, Italy; ^3^Clinical Unit of Physical Rehabilitation, IRCCS INRCA, Ancona, Italy; ^4^Unit of Geriatric Pharmacoepidemiology and Biostatistics, IRCCS INRCA, Cosenza, Italy; ^5^Unit of Neurology, IRCCS INRCA, Ancona, Italy; ^6^Department of Industrial Engineering and Mathematical Sciences, Polytechnic University of Marche, Ancona, Italy; ^7^Department Elderly Care, Diocesan Caritas Association for the Archdiocese of Cologne, Cologne, Germany; ^8^Universität Siegen, Wirtschaftsinformatik und Neue Medien, Siegen, Germany; ^9^Smart-Aging Research Center, Tohoku University, Sendai, Japan; ^10^Department of Rehabilitation Medicine, National Center for Geriatrics and Gerontology, Obu, Japan

**Keywords:** intrinsic capacity, active and healthy aging, functional ability, geriatrics, cognitive support, psychological support, multidomain, multicomponent intervention

## Abstract

**Introduction:**

The focus on intrinsic capacity (IC) could help clinicians to design interventions to improve the health of the older population. This review aims to map the current state of the art in the field of multi-domain interventions based on the IC framework, to allow health professionals in identifying personalized clinical interventions, oriented to empower the older people with a holistic and positive approach.

**Methods:**

A systematic review of the literature was conducted in July 2021 analyzing manuscripts and articles of the last 10.5 years from PubMed, Scopus, Embase, Google Scholar and Elsevier databases. A total of 12 papers were included.

**Results:**

The majority of successful interventions are based on a goal setting approach where the older people are involved in the definition of the strategy to follow to remain active and independent. None of the study have used the IC as a framework to design a clinical intervention.

**Conclusion:**

To the best of our knowledge, no other reviews are reported in the literature regarding the IC. Our study offers several research directions, which may take the existing debates to the next level.

## Introduction

Intrinsic capacity (IC) was defined as “the composite of all the physical and mental capacities of an individual” (1), including ability to walk, think, see, hear and re-member. Although older age is often characterized by a decline in baseline IC, the rate of decline widely varies among individuals and baseline IC reflects multiple setbacks and potential recoveries (2, 3). If some older adults are able to maintain functional in-dependence up to very advanced ages, other one’s experience early onset of severe functional disability which substantially affects their quality of life. According to WHO, such biological diversity can arise from inequity, understood as the differential influences of several factors including genetics, sex, ethnicity, and environment on aging itself (4). Anyway, progressive decline in IC may be more or less tolerated up to a critical point when individuals require care and support. IC is only one of many factors that determine biological age, but it can be an important focus for intervention to reduce the biological and functional age of older adults. Therefore, evaluation of bio-logical age through IC can enhance understanding of the functional trajectories and vulnerabilities of individuals and populations and guide individualized preventive measures and interventions that are tailored to the persons’ age, abilities and comorbidities (5).

Assessment of biological age through IC is of extreme importance for the future; losses of IC during the aging process may significantly affect quality of life and become manifested as common problems, such as hearing and vision impairments, memory loss, walking problems, urinary incontinence and loss of positive affect. For such impairments, older people often misbelief that there is no treatment available, and may then disengage from services, lack treatment adherence, with subsequent devastating effects on their quality of life. Recent studies have also shown that loss of IC may decrease quality of life and worsen prognosis in older adults (6). Moreover, IC decline was significantly associated with increased risk of frailty, disability, falls, fractures and death (7).

Regarding frailty and its connection with IC, Belloni et al. (8) assume that the two concepts can be seen as distinct but correlated points on a continuum in which IC represents the reserves of the individual on one side, while frailty is associated with the deficits accumulated with aging on the other. For this reason, it is essential to include also the concept of frailty in the assessment and analysis of the IC-driven interventions.

Due to the heterogeneity of the aging population, characterized by different levels of intrinsic capacity, personalized multicomponent health interventions may represent an effective way to promote health and subjective well-being achievements (9). However, to date, no systematic review focused on evidence about appropriate interventions to preserve intrinsic capacity and daily functioning in older individuals; for this reason, the aim of this systematic review is to map current state of the art in the field of multi-domain interventions based on the IC framework. The availability of evidence on multi-domain interventions that include the IC framework is essential to allow health professionals in identifying personalized clinical interventions, oriented to empower the older people with a holistic and positive approach.

## Materials and methods

### Literature search and study selection

The methodology of this systematic review was based on the Preferred Reporting Items for Systematic Reviews and Meta-Analyses (PRISMA) guidelines with the main aim of mapping the state of art of multi-domain interventions for older people, grounded on the IC framework. A systematic review of the literature was conducted in July 2021. The data were collected from PubMed, Scopus, Embase, Google Scholar and Elsevier databases, analyzing manuscripts and articles of the last 10.5 years (from January 2011 to June 2021), in order to obtain the latest evidence in the field. The PICOS format (P = population, I = interventions, C = comparator, O = outcome, S = study design) was adopted to formulate inclusion criteria. The inclusion criteria are as follows: (1) randomized controlled trials, quasi-experimental studies, or prospective or retrospective cohort studies, pre-post study with or without control groups; (2) testing of a multi-domain intervention to prevent or treat frailty in people aged ≥ 65 years; (3) classification in terms of (pre) frailty status according to an operationalized definition. Systematic and narrative reviews were excluded. A multi-domain intervention was defined as an intervention that intervenes in at least two different do-mains, including exercise therapy, nutritional intervention, hormone, cognitive or psychosocial interventions (10). As we refer to Intrinsic Capacity, we have included papers on multi-domain interventions on at least three areas within locomotion, cognitive, psychological, vitality and sensory.

Based on consultation with the multidisciplinary research team, multi-modal intervention studies were searched using the following search terms, and the combination thereof: olde*, elde*, intrinsic capacit*, functional ability*/functional status/functional trajectory*, healthy aging/successful aging, prefrail, virtual agent, coaching, self-management, multi-domain intervention, robotic*. The full search string is provided in Table 1.

**TABLE 1:** Search strategy.

Order of search	Terms
1	Olde* OR elde* AND “intrinsic capacit*”
2	Multicomponent OR multi-component OR multidimension* or multi-dimension*
3	1 AND 2
4	Olde* OR elde* AND “functional abilit*” OR “functional capacity” OR “functional status” OR “functional trajector*”
5	4 AND 2
6	Olde* OR elde* AND “Healthy aging”
7	6 AND 2
8	Olde* OR elde* AND “successful aging”
9	8 AND 2
10	Olde* OR elde* AND “active aging” OR “healthy aging” OR “successful aging”
11	10 AND 2
12	1 AND pre-frail
13	12 AND 2
14	1 AND virtual agent AND 2
15	1 AND coaching AND 2
16	1 AND self-management AND 2
17	1 AND multi-domain intervention AND 2
18	1 AND robotic* AND 2
19	Limit to English AND yr = 2011 -Current

yr, year. *Allows all words with the same root but different ending to be included.

After the preliminary search, 327,563 articles resulted from PubMed, 40,250 from Scopus, 40,098 from Embase, 91,898 from Google Scholar and 492,403 from Elsevier.

The findings were analyzed and screened by four experts of the team, a bioengineer, a clinical neuropsychologist, a statistician and a geriatrician. In particular, three review authors independently reviewed titles and abstract retrieved from the search in order to determine if they met the predefined inclusion criteria. The full text articles were subsequently analyzed.

The first screening was based on the analysis of the title of the findings. After the first step, 61 articles resulted from PubMed, 23 from Scopus, 16 from Embase, 33 from Google Scholar and 55 from Elsevier. A second screening was based on abstract analysis and deduplication of the findings. After this step 41 papers included from Pubmed, 18 from Scopus, 0 from Embase, 11 from Google Scholar and 33 from Elsevier. Another researcher (a statistician) confirmed the accuracy of the papers selection and screened for any possible omission.

### Data collection

**FIGURE 1:**
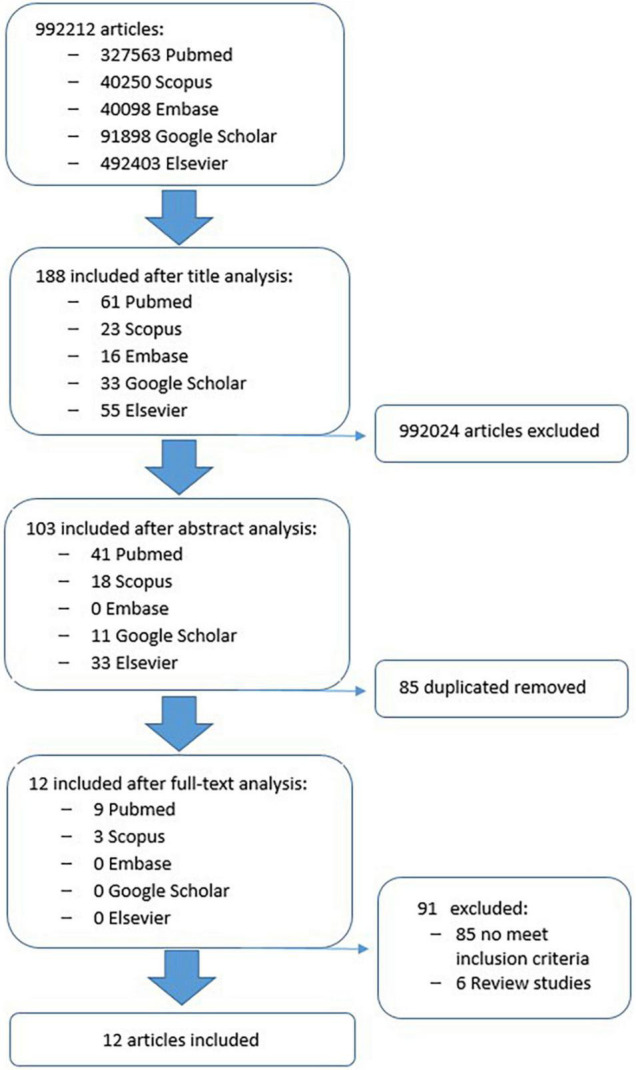
The flowchart search strategy.

After the screening based on the inclusion/exclusion criteria, conducted on the full text articles, the studies were selected as follows: 9 from PubMed, 3 from Scopus, 0 from Embase, 0 from Google Scholar, 0 from Elsevier database. Figure 1 shows the flowchart search strategy applied.

## Results

A total of 12 papers were included (11–22). The results could not be pooled into a meta-analysis due to clinical heterogeneity clearly observed in the participants’ involvement, in the type of intervention conducted, and in the outcome measures of the included studies.

### Study quality evaluation

Quality evaluation of 12 population-based studies was performed based on the PEDro scale, suggested for evidence-based reviews (23). The final score was settled when 3 authors reached agreement after repeated review and analysis. Of the twelve studies considered, the PEDro score ranged from 4 (12) to a maximum of 10 (11) (Table 2). In particular, 10 studies were rated as having a high (11, 13–22), 2 studies (12, 19) as having fair methodological quality.

**TABLE 2:** Scores of methodological quality assessment of the included studies.

PEDro	Ngandu et al., (11)	Scult et al. (12)	Clare et al. (13)	Ng et al. (14)	de Souto Barreto et al. (15)	Rainero et al. (16)	Moon et al. (17)	Huguet et al. (18)	Tabue-Teguo et al. (19)	de Souto Barreto et al. (20)	Kulmala et al. (21)	Lehtisalo et al. (22)
Eligibility	Y	Y	Y	Y	Y	Y	Y	Y	Y	Y	Y	Y
Randomized allocation	Y	N	Y	Y	Y	Y	Y	Y	N	Y	Y	Y
Concealed allocation	Y	N	Y	Y	Y	Y	Y	Y	N	Y	Y	Y
Baseline comparability	Y	Y	Y	Y	Y	Y	Y	Y	Y	Y	Y	Y
Blinded subject	Y	N	Y	Y	N	Y	N	N	N	N	N	N
Blinded therapists	N	N	N	N	N	N	N	N	N	N	N	N
Blinded raters	Y	N	Y	Y	Y	N	Y	N	N	N	N	N
Key outcomes	Y	Y	Y	Y	Y	Y	Y	Y	Y	Y	Y	Y
Intention to treat	Y	N	N	N	Y	N	Y	N	N	Y	N	Y
Comparison between groups	Y	N	Y	Y	Y	Y	Y	Y	Y	Y	Y	Y
Precision and variability	Y	Y	Y	Y	Y	Y	Y	Y	Y	Y	Y	Y
	10/11	4/11	9/11	9/11	9/11	8/11	9/11	7/11	5/11	8/11	7/11	8/11

*Y, yes; N, no.*

### General characteristics of the study population

All the studies were focused on older people with a mean age of 72.9 (± 5.5) years for the multi-domain intervention group and 73.2 (± 5.5) years in the control group. The number of participants involved in all the studies is 8,319, ranging from 46 to 1,827. There were 3,925 males and 4,394 females.

### Descriptive analysis and outcome measures

Table 3 shows the characteristics of the included studies. All studies evaluated the impact of multidimensional interventions on some domains of IC, but no one specifically focused on the IC framework as a whole entity. As regards evaluated domains, cognitive functioning was assessed by 7 studies (11, 13–15, 17, 19, 22), physical functioning by 6 studies (13–15, 18, 20, 21), vitality by 5 studies (13–16, 18), and psychosocial well-being by 6 studies (12–16, 18); none of the included studies has instead evaluated the sensory functioning.

**TABLE 3:** Descriptive analysis of the included clinical studies.

	Population		Intervention Type of Study	Measurements	Results
	Participants in multi-domain intervention group (MIG)	Participants in control group (CG)			
Ngandu et al., (11)	*n* = 591 older adults, 267 F/324 M Age: 69.5 ± 4.6 years	*n* = 599 older adults, 284 F/315 M Age: 69.2 ± 4.7 years	CG received regular health advice MIG group additionally received four intervention components: (1) nutritional guidance; (2) physical exercise; (3) cognitive training and social activity; and (4) intensive monitoring and management of metabolic and vascular risk factors Type of study: RCT	Primary outcome: Change in cognition as measured through comprehensive NTB Z score.	Estimated mean change in NTB total Z score at 2 years was 0⋯20 (SE 0⋯02, SD 0⋯51) in the MIG and 0⋯16 (0⋯01, 0⋯51) in the CG. Between-group difference in the change of NTB total score per year was 0⋯022 (95% CI 0⋯002–0⋯042, *p* = 0⋯030).
Scult et al. (12)	*n* = 46 older adults, 35 F/8 M Age: 75.5 ± 6.7 years	Not applicable	The intervention consisted of weekly, 90-min sessions for 9 consecutive weeks, directed by a psychologist. The program included sessions that taught participants: (1) a variety of methods to elicit the relaxation response, (2) the practice of adaptive coping and cognitions, (3) behaviors necessary to create a healthy lifestyle, and (4) methods of building social support. Type of study: pre- post- intervention analysis	Primary outcomes: Morale, measured through the PGCMS, CSES, a measure that addresses the multiple dimensions of self-efficacy.	The scores on both the PGCMS and the CSES increased significantly among completers of the intervention; i.e., the pre- to post intervention change was: (1) PGCMS, 1.68 ± 2.94, *p* = 0.001; (2) CSES, 33.90 ± 36. 30, *p* < 0.001. After the sensitivity analysis, the CSES pre-post change was still significant (*p* < 0.001), and the PGCMS trended toward statistical significance (*p* = 0.064)
Clare et al. (13)	Goal-setting group *n* = 24 older adults, 23 F/1 M Age = 67.50 ± 7.66 years Goal-setting with mentoring group *n* = 24 older adults, 19 F/5 M Age = 68.21 ± 7.92 years	*n* = 27 older adults, 23 F/4 M Age = 70.22 ± 7.77 years	In both the multi-domain intervention groups, participants engaged in a structured goal-setting process to identify up to five goals they wished to work on over the coming year relating to physical activity, cognitive activity, physical health and diet, and social engagement. Type of study: RCT	Primary outcomes: physical activity, assessed with the PASE, and cognitive activity, assessed with the FCAS. Secondary outcomes: psychosocial well-being, cognition, and physical health, fitness and diet.	Both the goal-setting and goal-setting with mentoring conditions increased their engagement in cognitive and physical activity. Changes in self-efficacy were negligible. Depression mean scores reduced in the control and goal-setting conditions, but increased in the goal-setting with mentoring condition. All three conditions improved in general cognitive ability assessed with the MoCA screening instrument. All three conditions reduced body fat percentage.
Ng et al. (14)	*n* = 96 older adults, 83 F/13 M Age: 75.61 ± 9.01 years	*n* = 98 older adults, 83 F/15 M Age: 77.90 ± 8.84 years	A bi-weekly program comprising cognitive training, physical-cognitive dual-task exercises and nutritional guidance was implemented. The program comprised 48 sessions (31% physical-cognitive dual-task exercises and 69% cognitive sessions) of which 19% were based on small group activities and 50% were computerized cognitive training. Nutritional guidance was intended to be on-going *via* the application throughout the length of the intervention. Type of study: RCT	Primary outcome: RBANS Secondary outcomes: EuroQol EQ-5D-5L, VAS, blood lipid panel and physical assessments.	There were no between-group differences in total RBANS score and domain scores after 6 months. There were also no between-group differences in quality of life measures and all blood parameters. here were no significant changes in total RBANS scores and immediate memory, visuospatial/constructional, language, and delayed memory scores in both the MIG and CG from baseline to follow-up. The MIG improved significantly in physical assessments.
Barreto et al. (15)	*n* = 60 older adults, 29 F/31 M Age: 75.2 ± 5.7 years	*n* = 60 older adults, 38 F/22 M Age: 73.2 ± 5.3 years	The web multi-domain platform focused on three lifestyles: nutritional advice, and exercise and cognitive training. The platform was equipped with a chat, to facilitate communication of participants with the research team, a personalized agenda showing the day-by-day activities (i.e., exercise and cognitive training to be done, nutritional advices), a library area where the content of the interventions and educational material on lifestyles were available Type of study: RCT	Primary outcomes: Feasibility and acceptability of study procedures and tools. Secondary outcomes: Cognitive function; physical function; depressive symptoms; nutritional status; HRQOL; Physical Activity; Leisure-time cognitive activities; Food intake	Regarding feasibility, 58 (out of 60) participants in MIG connected to the multi-domain platform at least once during the 6-month trial. Regarding acceptability, 7.5% said the platform was not ready to be used and needed major changes; 5.7% indicated it required minor changes; 34% said it was ready to be used, but minor modifications; and 52.8% indicated the platform was ready to be used without any change. No statistically significant effects were found, except for the two variables of HRQOL, showing MIG had an improved HRQOL compared to CG.
Rainero et al. (16)	*n* = 101 older adults, 71 F/30 M Age: 70.37 ± 6.15 years	*n* = 100 older adults, 77 F/23 M Age: 73.40 ± 6.57 years	Intervention packages were developed for physical, cognitive, psychosocial, nutrition and sleep domains. Type of study: RCT	Primary outcomes: QoL; mood and nutrition function.	CG displayed a significant decrease in QoL at the 12-month phase, with no change in QoL evident in MIG. MIG displayed a significant increase in nutrition score at the 12-month phase relative to the 6-month phase
Moon et al. (17)	Facility-based MI (FMI) *n* = 48 older adults, 35 F/13 M Age = 71.6 ± 4.8 years Home-based MI (HMI) *n* = 50 older adults, 36 F/14 M Age = 70.9 ± 5.0 years	*n* = 42 older adults, 33 F/9 M Age = 70.1 ± 4.6 years	The 24- week intervention comprised vascular risk management, cognitive training, social activity, physical exercise, nutrition guidance, and motivational enhancement. The FMI participants performed all intervention programs at a facility three times a week. The HMI participants performed some programs at a facility once every 1–2 weeks and performed others at home. Type of study: RCT	Primary outcomes: Feasibility measured through retention, adherence, and at least no differences from the CG in the RBANS	The retention rates were 88.2% and 96.1%, and adherence to the intervention was 94.5% and 96.8%, respectively. The RBANS total scale index score improved significantly in the FMI (5.46 ± 7.50, *P* = 0.004) and HMI (5.50 ± 8.14, *P* = 0.004) groups compared to the control group (−0.74 ± 11.51).
Huguet et al. (18)	*n* = 100 older adults, 68 F/32 M Age: 84.5 ± 3.4 years	*n* = 100 older adults, 61 F/39 M Age: 84.5 ± 3.7 years	6-month multifactorial intervention was based on four axes: (1) Assessment of inadequate prescription in polypharmacy patients. (2) Group session, led by an expert on the Mediterranean diet. (3) Physical exercise program. (4) Review of personal and environmental conditions and social support. Type of study: RCT	Primary outcomes: Frailty, Functional and nutritional status, adherence to Mediterranean diet, quality of life, and functional mobility.	Frailty was lower in the intervention group (RR 2.90; 95%CI 1.45–8.69). Functional and nutritional status, adherence to Mediterranean diet, quality of life, and functional mobility were improved in MIG (*p* ≤ 0.001).
Barreto et al. (20)	*n* = 816 older adults, 534 F/282 M Age: 75.3 ± 4.3 years	*n* = 821 older adults, 525 F/296 M Age: 75.3 ± 4.5 years	The MAPT intervention was composed of 3 main components: cognitive training (memory and reasoning), nutrition counseling, and advice on physical activity. Twelve 2-h sessions (1 h of cognitive training, 45 min of advice on physical activity, and 15 min of nutrition counseling) were provided in the first 2 months of the study, followed by a 1-h session each month until the end of the 3-year study. Type of study: RCT	Primary outcomes: severity of frailty (continuous FI score), incident frailty, incidence of persistent frailty (frailty at 2 consecutive time points), and reversibility of frailty (from frailty to non-frailty)	MIG had a decreased risk of developing both frailty (hazard ratio 0.72; 95% confidence interval, 0.55–0.93) and persistent frailty (hazard ratio 0.53; 95% confidence interval, 0.33–0.85).
Kulmala et al. (21)	*n* = 631 older adults, 286 F/345 M Age: 69.7 ± 4.6 years	*n* = 629 older adults, 303 F/326 M Age: 69.4 ± 4.7 years	The FINGER multi-domain intervention included simultaneous physical activity intervention, nutritional counseling, vascular risk monitoring and management, and cognitive training and social activity. Type of study: RCT	Primary outcomes: The ability to perform daily activities (ADLs and instrumental ADLs) and physical performance (Short Physical Performance Battery).	The difference in the change between MIG and CG was −0.95 (95% CI = −1.61 to −0.28) after 1 year and −1.20 (95% CI = −2.02 to −0.38) after 2 years. MIG had a slightly higher probability improvement (from score 3 to score 4; *P* = 0.041) and a lower probability of decline (from score 3 to scores 0–2; *P* = 0.043) for physical activity compared to CG.
Lehtisalo et al. (22)	*n* = 571 older adults, 263 F/308 M Age: 69.5 ± 4.6 years	*n* = 584 older adults, 278 F/306 M Age: 69.1 ± 4.7 years	The FINGER multi-domain intervention included simultaneous physical activity intervention, nutritional counseling, vascular risk monitoring and management, and cognitive training and social activity. Dietary intervention was combination of individual counseling (3 sessions) and group meetings (6 sessions), mainly during the first year Type of study: RCT	Primary outcome: Cognitive performance Secondary outcomes: cognitive domain Z scores for executive function; processing speed; and memory domain.	Adherence to healthy diet at baseline predicted improvement in global cognition, regardless of MIG (*P* = 0.003). Dietary improvement was associated with beneficial changes in executive function, especially in MIG (*P* = 0.008; *P* = 0.051 for groups combined).
Tabue-Teguo et al. (19)	No Frailty group: *n* = 799 older adults, 509 F/290 M Age = 74.41 ± 4.00 years Frailty group: *n* = 665 older adults, 431 F/234 M Age = 76.32 ± 4.62 years	Not applicable	The MAPT intervention consisted of 2 h’ group sessions focusing on three domains (cognitive stimulation, physical activity, and nutrition) and a preventive consultation (at baseline, 12 months, and 24 months). For Omega-3 Polyunsaturated Fatty Acids supplementation, participants took two capsules of either placebo or polyunsaturated fatty acids daily. Type of study: comparison between groups	Primary outcomes: Change in cognitive tests over 36 months	No differences in the change in cognitive tests over 36 months. A trend toward significance difference in TMT-A (*P* = 0.031) were found for the effect of the multi-domain intervention between the two groups.

*n, number of subjects; F, female; M, male; MIG, Multidomain Intervention Group; CG, Control Group; RCT, Randomized Controlled Trial; NTB, Neuropsychological Test Battery; PGCMS, Philadelphia Geriatric Center Morale Scale; CSES, Self-efficacy the Coping Self-Efficacy Scale; PASE, Physical Activities Scale for the Elderly; FCAS, Florida Cognitive Activities Scale; MoCA, Montreal Cognitive Assessment; RBANS, Repeatable Battery for the Assessment of Neuropsychological Status; VAS, Visual Analog Scale; HRQOL, health-related quality of life; QoL, Quality of Life; ADLs, activities of daily living; CI, confidence interval.*

### Intervention effects

Below is a brief description of the main results reported in the 12 population-based studies categorized according to domains of intervention.

#### Cognitive functioning

Seven out of twelve studies assessed the effects of interventions on cognitive functioning (11, 13–15, 17, 19, 22). In six studies (11, 14, 15, 17, 19, 22) the intervention was represented by a multidomain training addressing some or all IC domains and made of several components, such as physical activity, cognitive training and social activity, nutritional advice, monitoring and management of risk factors; in one study (13), the multidimensional intervention was designed through a goal-setting approach, asking patients to set up to five goals they wished to accomplish within the coming year.

Four (11, 17, 19, 22) out of the six studies using multidomain intervention training (11, 14, 15, 17, 19, 22) showed a significant benefit on individual cognitive function over time. In particular, in the study by Ngandu et al. (11), individuals in the intervention group (IG) underwent a significant improvement of cognitive scores, executive function and processing speed, even after 2 years; similarly, Moon et al. (11) showed benefits of a 24-week multidomain training on cognitive scores; in the study by Tabue-Tego et al. (19), despite cognitive tests were not significantly different between the control group (CG) and the IG after 36 months of follow-up, a significant trend toward improvement in Trail Making test part A (TMT-A) performance was found in the IG; finally, Lehtisalo et al. (22) showed how adherence to nutritional guidelines in the context of a multidimensional intervention led to benefits in terms of global cognition and executive functioning; only one study (13) assessed the usefulness of a goal-setting approach; individuals in the IG were asked to set up to five goals to accomplish within the coming year; individuals following the goal-setting approach were divided in two groups according to the presence of bi-monthly telephone mentoring. The two goal-setting groups increased their level of cognitive activity relative to controls and achieved additional benefits compared to control in memory and executive function. Adding follow-up mentoring produced further benefits compared to goal-setting alone in global cognition and memory.

Finally, two studies (14, 15) showed no significant effects of multidomain interventions on cognitive scores during follow-up.

#### Physical activity

Six out of twelve studies assessed the effects of interventions on physical functioning (13–15, 18, 20, 21). In five studies (13–15, 18, 20, 21), the intervention was represented by a multidimensional training program, while in the study by Clare et al. (13) a goal-setting approach was used. Most of the studies reported beneficial effects of multidomain interventions on physical function over time (13, 14, 18, 20, 21), whereas one study (15) showed no significant effect.

In the pilot study by Clare et al. (13), the goal-setting approach with or without mentoring was associated with improved engagement in physical activity, as well as flexibility, grip strength, balance, and agility; furthermore, the goal-setting approach with mentoring improved physical fitness compared to goal-setting approach without mentoring.

In the study by Ng et al. (14), a 24-week multi-domain intervention for older adults at risk of cognitive impairment at neighborhood senior centers was implemented. The program comprised dual-task exercises, cognitive training, and mobile application-based nutritional guidance. Patients in the IG underwent an improvement in Chair Stand Test and grip strength after 24 weeks. Similarly, in the study by Huguet et al. (18), potential benefits of a multidimensional training program were evaluated among 200 community-dwelling pre-frail older patients; at 12 months, individuals in the IG were characterized by lower prevalence of frailty and improved function mobility, with better performance in both the Timed Up and Go (TUG) and Five Time Sit to Stand (FTSST) tests. In the secondary analysis of the MAPT study by de Souto Barreto et al. (20), the effects of a long-term (3-years) multi-domain lifestyle intervention on the severity and incidence of frailty in older adults was investigated. Compared with controls, subjects in the multi-domain group had a decreased risk of developing both frailty and persistent frailty.

Another important study aimed to investigate the effect of multi-domain lifestyle intervention on daily functioning of older people was the Finnish Geriatric Intervention Study to Prevent Cognitive Impairment and Disability (FINGER) conducted by Kulmala et al. In their first publication (21) they analyzed, for 2 years, a total of 1,260 older adults who were at risk of cognitive decline. The multi-domain intervention included simultaneous physical activity intervention, nutritional counseling, vascular risk monitoring and management, and cognitive training and social activity. During the 2-year intervention, the activity of daily living (ADL) disability score slightly increased in the control group, while in the intervention group, it remained relatively stable. In terms of physical performance, the intervention group had a slightly higher probability of improvement and a lower probability of decline for chair rise compared to the control group.

As previously reported, only one study (15) showed no benefit of multidomain intervention on physical activity. In this 6-month eMIND project by Barreto et al. (15), researchers evaluated the effects of a multi-domain lifestyle intervention composed of cognitive training, exercise training, and nutritional advices among community-dwelling older adults. One hundred-twenty participants were enrolled and randomized in the multi-domain intervention group and control group. Compared to controls, the intervention had a positive effect on health-related quality of life; no significant effects were observed across the other clinical and lifestyle outcomes.

#### Vitality

Five out of twelve studies assessed the effects of multi-intervention training on vitality (13–16, 18). Explored aspects of this domain included physical health (13–15, 18, 21), nutritional status (13, 15, 16, 18), and laboratory parameters (13, 14). In four studies (14, 15, 18, 21), the intervention was represented by a multidimensional training program, while in the study by Clare et al. (13) a goal-setting approach was used. Most of the studies reported beneficial effects of multidomain interventions on vitality at follow-up (13, 14, 18, 21), whereas one study (15) showed no significant effect.

The goal-setting approach (13) was associated with increased physical health, as measured in terms of aerobic capacity, flexibility, balance, agility, and hand grip strength; such approach was also associated with decreased serum cholesterol levels and decreased body fat percentage. Similarly, several multidomain trainings resulted to add some benefit in the IG; observed benefits included increased hand grip strength (14), preservation of daily functioning assessed *via* ADL (21), and increased nutrition and adherence to healthy diet habits (16, 18).

#### Psychosocial well being

Six out of twelve studies assessed the effects of multi-intervention training on psychosocial well-being (12–16, 18). Several aspects of this domain were investigated: self-efficacy and morale (12), mood (12, 13, 15), quality of life perception (14–16, 18), engagement in social and leisure activities (15). Most of the studies reported beneficial effects of multidomain interventions on psychosocial well-being (12, 13, 15, 16, 18), whereas only one study (14) showed no significant benefits.

In the study by Scult et al. (11), the researchers evaluated the effect of a healthy aging program for older adults on self-efficacy and morale. The Mind Body Intervention consisted of weekly, 90-min sessions for 9 consecutive weeks, directed by a psychologist. The program included sessions that taught participants: (1) a variety of methods to elicit the relaxation response, (2) the practice of adaptive coping and cognitions, (3) behaviors necessary to create a healthy lifestyle, and (4) methods of building social support. Significant increases in self-efficacy and morale were observed for program completers. In the study by Clare et al. (13), the goal-setting approach was associated with decreased depression scores, whilst changes in self-efficacy among groups were negligible.

Effects of multidimensional interventions on quality-of-life improvement were largely confirmed; in the study by Barreto et al. (14), HRQoL was the only dimension to improve in patients belonging to IG compared to CG; similarly, Rainero et al. (16) showed the effects of multidimensional interventions in preserving quality of life of pre-frail older adults after 12 months of follow-up; additionally, active participants showed an increase in mood during the follow-up period; furthermore, Huguet et al. (18) demonstrated a net improvement in quality of life perception among participants undergoing a 6-month four-dimensional intervention.

The secondary analysis of the MAPT study was conducted by de Souto Barreto et al. (20) to investigate whether a long-term (3-years) multi-domain lifestyle intervention was associated with the severity and incidence of frailty in older adults. Authors recruited 1,637 older people divided in 821 controls and 816 who received a multi-domain lifestyle intervention (cognitive training, nutrition counseling, and advice on physical activity). The intervention involved 12 2-h sessions (in the first 2 months) followed by a 1-h session each month until the study end. Controls received the usual care but did not receive any personalized lifestyle intervention. The 4 outcomes were severity of frailty (continuous FI score), incident frailty, incidence of persistent frailty (frailty at 2 consecutive time points), and reversibility of frailty (from frailty to no-frailty). Compared with controls, subjects in the multi-domain group had a decreased risk of developing both frailty and persistent frailty.

Another important study aimed to investigate the effect of multi-domain lifestyle intervention on daily functioning of older people is the Finnish Geriatric Intervention Study to Prevent Cognitive Impairment and Disability (FINGER) conducted by Kulmala et al. In their first publication (21) they analyzed, for 2 years, a total of 1,260 older adults who were at risk of cognitive decline. The multi-domain intervention included simultaneous physical activity intervention, nutritional counseling, vascular risk monitoring and management, and cognitive training and social activity. During the 2-year intervention, the activity of daily living disability score slightly increased in the control group, while in the intervention group, it remained relatively stable. In terms of physical performance, the intervention group had a slightly higher probability of improvement and a lower probability of decline for chair rise compared to the control group.

The same data have been used by Lehtisalo et al. (22) to evaluate the effect of dietary changes adopted in older age. Adherence to healthy diet at baseline predicted improvement in global cognition, regardless of intervention allocation. Dietary improvement was associated with beneficial changes in executive function, especially in the intervention group.

## Discussion

In the past, the study of aging process was strongly focused on health deficits (8), such as diseases, disabilities, and limitations; this view was supported by the strong relationship between increase in socio-economic burden on healthcare systems world-wide and the increase in prevalence of multimorbidity and disability among populations with high life expectancy. Despite the relevance of this model, aging should be investigated more broadly, since absence of diseases does not always go hand-in-hand with aging well. Rather than considering healthy aging from the disease-based perspective, the functioning-based approach promoted by WHO is oriented around building and maintaining the ability of older people to be and to do the things they have reason to value (4).

The availability of evidence on multi-domain interventions that include the IC framework is of paramount relevance for the health professionals, as they may provide them useful personalized strategies, to support the older patients’ resilience and autonomy in daily life, and can be easily integrated with more traditional therapies and treatments. From the analysis of the selected multi-domain interventions, there are important considerations that can be taken into account.

First of all, the majority of successful interventions are based on a goal setting approach (24, 25): the older people are involved in the definition of the strategies to follow to remain active and independent. The wellbeing of the elderly does not necessarily fit the intervention goals derived from the prevention perspective of the researcher. It is very important to include the elderly themselves in the goal-setting process, as they prefer to set goals to achieve well-being that are more focused on the process of adaptation to any functional loss (26). However, a balance between personalization and clinical effectiveness should be reached in agreement with the participants, before the testing phase, in order to find a minimum core of standardized strategies to complement the personalized approach. This may allow the comparability and replicability of the intervention, in addition to assure the adherence and the compliance of the older people.

Despite the undeniable wealth of the IC framework, none of the study have used this to design the intervention, but only to assess the improvement in IC domains. From the analysis of the studies, Physical Activity is the domain that has been received the most of the attention, including specific multicomponent interventions to improve different functional capabilities such as aerobics, muscle strength, balance and gait, while Psychological support has been addressed mostly as counseling activity through pre-selected contents instead of a more patient-centered approach. How to include sensory domain still represents an open topic for the studies in the field. Within the clinical outcomes, moreover, self-efficacy and goal attainment should be considered as important psychosocial determinants to be assessed after any multi-domain intervention previous studies have shown that self-efficacy and social support in older women enhances adherence to strength-training programs (27). In the meta-analysis, barrier self-efficacy was involved in the maintenance of exercise behavior (28). Those competences, in fact, are drivers for the improvement of the health status, as well as for IC and functional ability maintenance (29). They also constitute the basis for the adoption of healthy lifestyles, assuring the sustainability of positive behaviors in the long-term (30). Nevertheless, an assessment tool to identify improvement of Intrinsic Capacity as a whole, not only as sum of domains, is still missing.

In order to be effective, any intervention should be adapted to the older people, easily accessible and integrated into the everyday life (31). At this purpose, the field of coaching through technology is receiving more and more interest, as effective strategies to provide patient-centered multicomponent healthcare interventions integrated with technology to foster self-management, prevention, adherence to treatments, positive health outcomes, and resilience, all factors that improve the IC (32). Therefore, the relevance of this study stands also in the way to identify the existing research trends and possible gaps that need to be applied in the near future when designing technology-based interventions. Indeed, all the aforementioned key strategies (i.e., the goal set-ting approach, the involvement of older adults in the definition of the strategy to follow to remain active and independent, etc.), could be seen as interconnectors between the field of technologies and the IC.

Despite this positive aspect, there are some limitations to this review. Firstly, data sources were drawn from specific databases (i.e., PubMed, Scopus, Embase, Google Scholar and Elsevier). The choice of using specific search terms could have omitted some results from the search. Moreover, we collected a relatively small sample of studies and excluded non-English language studies. It could be possible that other literary sources were available in other unselected databases or in other languages. Another possible limitation is the average age of the patients included in the studies analyzed, which is rather low and refers to an audience of young old people. Therefore, the conclusions we reached cannot be transferred to the entire elderly population. Moreover, results obtained should be interpreted with caution because some studies included in the review were reported as being built with low methodological quality. Despite these limitations, our study offers several research directions, which may take the existing debates to the next level.

## Data availability statement

The original contributions presented in the study are included in the article, further inquiries can be directed to the corresponding author/s.

## Author contributions

EM and RB: study concept and design. EM, RB, SC, and ET: acquisition of data (literature search and study selection). EM, RB, LS, and ET: analysis and interpretation of data (literature). EM, RB, LS, and VS: writing—original draft preparation. FL, GR, AC, and GP: critical revision of the manuscript for important intellectual content. JM, RW, TO, SW, KK, and IK: supervision. JM and TO: writing—review and editing. All authors contributed to the article and approved the submitted version.

## Conflict of interest

The authors declare that the research was conducted in the absence of any commercial or financial relationships that could be construed as a potential conflict of interest.

## Publisher’s note

All claims expressed in this article are solely those of the authors and do not necessarily represent those of their affiliated organizations, or those of the publisher, the editors and the reviewers. Any product that may be evaluated in this article, or claim that may be made by its manufacturer, is not guaranteed or endorsed by the publisher.
